# Muscle strength assessment of critically ill patients is associated with functional ability and quality of life at hospital discharge

**DOI:** 10.1186/cc11136

**Published:** 2012-03-20

**Authors:** G Sidiras, I Patsaki, M Dakoutrou, E Karatzanos, V Gerovasili, A Kouvarakos, A Kardara, K Apostolou, S Dimopoulos, V Markaki, S Nanas

**Affiliations:** 1 University of Athens, Greece

## Introduction

Patients with critical illness after hospital discharge often exhibit poor functional ability and quality of life as a consequence of acquired muscle weakness. The Medical Research Council (MRC) strength score and hand-grip dynamometry (HGD) are reliable and valid methods to detect clinically significant muscle weakness. The objective of this study is to examine the correlation of these instruments to functional ability and quality-of-life questionnaires at hospital discharge.

## Methods

Two hundred and sixty-six consecutive patients who had been discharged from the ICU were evaluated and 37 of them were eligible (inclusion criteria: in mechanical ventilation >72 hours, a cognitive status that allows assessment) for the study (mean ± SD: age 55 ± 15; APACHE 14 ± 5; SOFA 8 ± 3; length of ICU stay 22 ± 22 days; duration of mechanical ventilation 17 ± 19 days). Muscle strength was evaluated with the MRC score and HGD every 7 days until discharge from the hospital. The Functional Independence Measure (FIM) was used to evaluate the functional ability while health-related quality of life was assessed by the Nottingham Health Profile (NHP).

## Results

At hospital discharge the MRC scale and HGD were significantly correlated with FIM (*r *= 0.69, *P *< 0.001 and *r *= 0.58, *P *< 0.001, respectively). There seems to be a good correlation of the MRC scale (*r *= -0.57, *P *< 0.001) with the section of mobility of the NHP. There is also certain association among the domain of mobility and energy of the NHP with the FIM (*r *= -0.88, *P *< 0.001 and *r *= -0.61, *P *< 0.05, respectively).

## Conclusion

The significantly reduced muscle strength of critically ill survivors could have detrimental effects on their mobility and quality of life. By this study it was shown that muscle strength assessment was well associated with functional ability. We assume that this might be a possible significant prognostic role.

